# Automated procedure to assess pup retrieval in laboratory mice

**DOI:** 10.1038/s41598-022-05641-w

**Published:** 2022-01-31

**Authors:** Carmen Winters, Wim Gorssen, Victoria A. Ossorio-Salazar, Simon Nilsson, Sam Golden, Rudi D’Hooge

**Affiliations:** 1 Laboratory of Biological Psychology, University of Leuven (KU Leuven), Leuven, Belgium; 2 Leuven Experimental Attachment Research Lab, KU Leuven, Leuven, Belgium; 3 Department of Biosystems, Center for Animal Breeding and Genetics, KU Leuven, Leuven, Belgium; 4 Department of Biological Structure, University of Washington, Seattle, WA USA

**Keywords:** Social behaviour, Network models

## Abstract

All mammalian mothers form some sort of caring bond with their infants that is crucial to the development of their offspring. The Pup Retrieval Test (PRT) is the leading procedure to assess pup-directed maternal care in laboratory rodents, used in a wide range of basic and preclinical research applications. Most PRT protocols require manual scoring, which is prone to bias and spatial and temporal inaccuracies. This study proposes a novel procedure using machine learning algorithms to enable reliable assessment of PRT performance. Automated tracking of a dam and one pup was established in DeepLabCut and was combined with automated behavioral classification of “maternal approach”, “carrying” and “digging” in Simple Behavioral Analysis (SimBA). Our automated procedure estimated retrieval success with an accuracy of 86.7%, whereas accuracies of “approach”, “carry” and “digging” were estimated at respectively 99.3%, 98.6% and 85.0%. We provide an open-source, step-by-step protocol for automated PRT assessment, which aims to increase reproducibility and reliability, and can be easily shared and distributed.

## Introduction

Maternal care determines an infant’s physical and functional development^1–4^. Discriminative maternal care during the postpartum period is ensured by the formation of some sort of mother-infant bond^4,5^. Human studies that investigate maternal care and its many effects on infant development are complicated by a variety of practical and ethical factors, lack of control over environmental and genetic background, inaccessibility of brain samples^6^. Animal models, particularly rodents, provide a valid research tools as many neural and hormonal mechanisms of care and bonding are homologous between mammalian species^6,7^.

The Pup Retrieval Test (PRT) is the most widely used assay to assess maternal care in fundamental and preclinical rodent research^8^. In its most basic design, it quantifies the mother’s retrieval response to the removal of a pup from the nest, a sequence of pup-directed sensorimotor responses elicited by (multi)modal stimuli from the infant, and processes by the mother^4,9–13^. The test has been used to study the impact of pharmacological and environmental interventions on maternal care^14–18^. Finally, it has been used in research on disorders that affect mother-infant interaction, such as autism, fetal alcohol syndrome, chronic prenatal stress, postpartum depression, schizophrenia^19–24^.

It was established that differences in methodology, environment and experimenter intervention influence behavior and its assessment^25^. Typical PRT protocols rely on manual scoring, which provides only basic information and is prone to spatial and temporal inaccuracies as well as bias^26^. Some authors resorted to limited end-point registration to simplify manual PRT scoring^27,28^. Components such as “maternal latency to start retrieving the pup”, “time necessary to complete retrieval”, and “efficacy of the retrieval” have been scored during real-time observation or from video recordings, but pup retrieval is a dynamic, interactive behavior that contains more information than these variables^28^. Also, scoring these and other components from videos, or with video tracking software, requires laborious indexing of the behavioral components on a frame-by-frame basis^29,30^. Scoring programs may not allow such frame-based analyses, and scoring tends to be a slow and laborious process. Furthermore, manual analyses require well-defined rules to define start, duration and end of behavioral components^31^. These rules are researcher and context specific, which affects transferability between laboratories^29,30,32,33^. Further, temporal accuracy is especially relevant for PRT behaviors that are triggered by pup ultrasonic vocalizations and have a millisecond profile^9^.

Precise and standardized recording of the maternal response would be most reliably achieved by automated procedures^34^. Manual PRT scoring is often inaccurate, involving subjective judgement of the completion of a retrieval event, imprecise definition of nest borders, etc. We therefore present a novel procedure using machine learning algorithms and open-source software to enable reliable, automated PRT registration. Recent advances in motion capture and deep-learning allow extraction of behavioral variables from recorded videos without elaborate recording hardware^35^. Tracking and behavioral analysis tools reach at least human-like accuracy, and even outperform manual scoring with unprecedented rapidity^27,36^. We established automated tracking of a dam and one pup using DeepLabCut software, and classified variables such as “maternal approach”, “carrying” and “digging” using Simple Behavioral Analysis (SimBA). Specifically, this paper aims to (1) introduce a dataset for a dam-pup PRT tracking network, including operational definitions and underlying rationale; (2) establish SimBA classification of ethologically relevant PRT behaviors; (3) show that an automated procedure is able to quantify PRT accurately; (4) create a SimBA add-on to evaluate PRT results using an easy-to-use graphical user interface; (5) provide a user-friendly, step-by-step protocol to replicate or expand the present study. All annotated images and videos, tracking models and behavioral classifiers are available at 10.17605/OSF.IO/RWHTD.

## Results

### Video pre-processing

C57BL6J mice were subjected to the PRT on postnatal day 5 (P5). Videos were recorded in the home cage using a Foscam C2 IP-camera (EUport, Wageningen) from an overhead camera perspective (top-down). Per dam, one video file was obtained containing six single pup retrieval trails. This one overall video was splint into six single pup trial videos. To decrease file size and increase efficient neural network training, videos were cropped around the top edges of the home cage and grey-scaled.

### Tracking dataset and training

**Figure 1:**
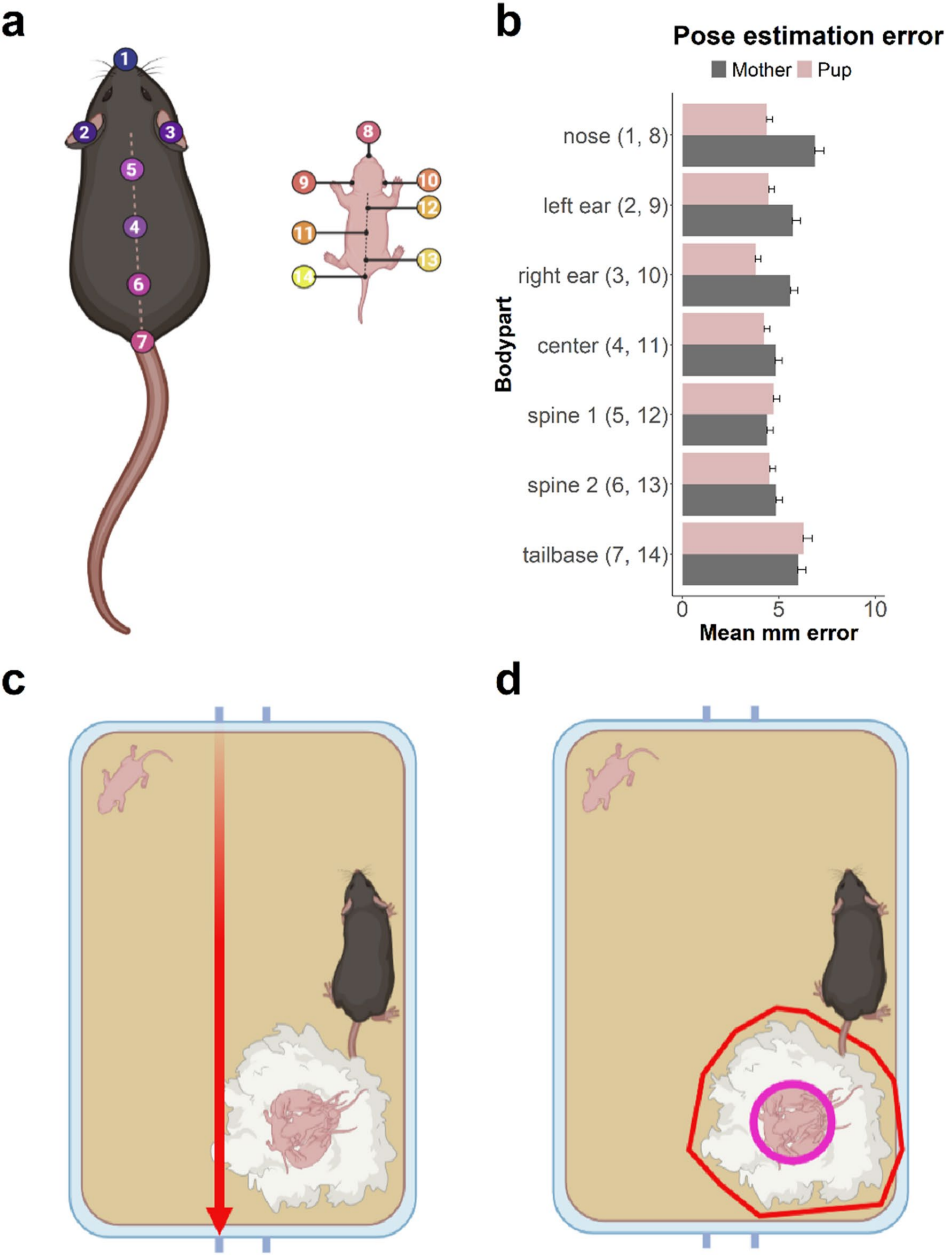
Pose estimation and region of interest (ROI) definition. (**a**) Schematic representation of the 14-body part pose configuration used for the PRT protocol in DeepLabCut (image was created with BioRender.com). (**b**) Mean millimeter error per body part and subject for the DeepLabCut PRT pose estimation model. Means were calculated without the use of an estimation p-cutoff. In Supplementary File 2 Table S2, the mean mm errors are shown with different p-cutoff values. (**c**) Illustration of distance on a Type-II mouse cage. We used the length between opposite cage tag hinges as standard length. The pink line indicates a distance of 267 mm. (**d**) Example of region of interest (ROI) definition in SimBA. The yellow area indicates the nest site and the pink circular area the core nest. The red markers do not display any ROI, but indicate that the top corners of the home cage need to be visible in the cropped video.

DeepLabCut 2.1.10.4 (DLC^35^) was used to create a dam-pup tracking algorithm in the pup retrieval protocol. 592 Frames were randomly extracted from 38 single trial videos, using the k-means algorithm in DLC. Next, 14 body parts were manually annotated with high stringency, with seven body parts on the dam and seven on the isolated pup (see Fig. 1a). Occluded body parts in the established training dataset were simulated by using the body part configuration from a similar frame in which the occluded animal was visible as a template. The operational definitions of these body parts are shown in Supplementary File 1 Table S1.

The dam-pup network was trained in Google Colaboratory (https://colab.research.google.com/) using the ResNet-50 architecture in DeepLabCut with a batch size of 4 images. A 95:5 train/test ratio was used, meaning that 95% of the 592 annotated frames was used to train the neural network and 5% is used to evaluate network performance. Training of neural networks means that parameters of a mathematical model is iteratively optimized to increase prediction accuracy on the training dataset. As the aim of the dam-pup model is to generalize well in the future, the model should not be overfitted. Overfitted models will only “memorize” initial data meaning they will perform poorly on unseen videos recorded with slightly different animals, lighting or camera distance^37^. Therefore, training was stopped after 70,000 iterations as optimization minimized and model parameters of every 1000th iteration were stored.

### Performance evaluation of the trained network

Network performance was evaluated by the computation of a train and test error as measured by the average Euclidian difference between the pixel coordinates from manual annotations and DLC estimations. Prior to selection, four criteria were defined to evaluate the best performing model. First, the model should not be overfitted and thus the best performing model with the lowest number of iterations should be chosen. Second, mean test and train error over all body parts should be as low as possible. Third, especially maternal nose and ears should have a low mean error as these will be important for the behaviors in the PRT. Fourth, the nest can occlude body parts and consequently impact tracking accuracy. Thus, mean Euclidian errors should decrease after increasing the likelihood threshold and thus calculating mean test or train errors for DLC estimations above a defined probability. The best performing dam-pup model was created after 50,000 iterations. The estimated error averaged over all 14 body parts was 2.3 pixels (px) for the training dataset and 10.44 px for the test dataset (Fig. 1b). The mean Euclidian errors per body part and using different likelihood thresholds are shown in Supplementary File 2 Table S2.

### Distance standardization and definition of Regions of Interest

Euclidean pixel distances were standardized to millimeter distances using Simple Behavioral Analysis (SimBA^31^). Here, the length between the top lids of the home cage (Fig. 1c) is used to define a standardized distance of 265 mm. The mean errors per body part were standardized to a metric scale and ranged between 2.2 and 4.5 mm as shown in Fig. 1b. Hereafter, per video two regions of interest were defined in SimBA: the nest and the core nest. The nest is defined as a polygonal that encloses the entire nesting site (Fig. 1d, red), whereas the core nest is defined as a circle that encloses only the site of the nest in which the pups are (Fig. 1d, pink).

### Quality control of DeepLabCut tracking output

Data are tracked from DLC in SimBA to train behavioral classifiers. Since tracking inaccuracies (such as impossible locations or movements) can complicate classifier training, they should be corrected. Outliers were defined by using the median distance between nose and spine1: median distance multiplied by 2.5 was the criterion to define movement outliers; median distance multiplied by 4 defined location outliers.

### Random forest classifiers

SimBA was used to develop three random forest models to predict maternal approach, maternal carrying and maternal digging. In total, 27,683 frames of seventeen videos were manually annotated using the SimBA event logger for the absence (‘0’) or presence (‘1’) of each of the three behaviors. A subset of nine videos was used to train the approach and carry models, while the digging model was built using ten videos, yielding respectively 10,157 and 18,207 frames (Table 1). All three random forest models were trained by creating 2000 decision trees from the training data. Frames containing the behaviors of interest were imbalanced compared to frames absent of these behaviors. Therefore, the majority class (i.e. absent behavior) was randomly under-sampled at ratios shown in Table 1. Based on precision-recall curves, an optimal classifier performance was chosen on the basis of f1. To balance the number of false-positive and false-negative classifications, we chose the discrimination threshold at the highest f1 value. These discrimination thresholds and minimum bout lengths can be found in Table 1.

**Table 1:** Summary statistics on data used for behavioral classification.

Classifier	# Annotated frames	Behavior present [%]	Random under-sample ratio	Test set: frames present	Test set: frames absent	Discrimination threshold	Minimum bout length (ms)
Approach	10,157	8.7	8.5	604	5302	0.47	500
Carry	10,157	13.9	16	828	4443	0.47	200
Digging	18,207	32.3	2	930	1802	0.24	1000

### Performance of random forest classifiers

After training, an estimation was made for the model which estimates the difference between the original input and the predicted output. This, however, does not give an indication of the translatability of the model to an independent dataset. Here, seventeen videos were manually annotated frame-by-frame and were joined into an independent dataset. Using the Caret package in R^38^, the performance of the three classifiers was evaluated.

The independent dataset for carry and approach consisted of 17,526 frames (approach present = 13.9%, carry present = 8.7%; Table 1). Confusion matrices for carry, approach and digging behavior are shown in Table 2. The approach and carry classification accuracy was respectively 98.6% (95% CI 98.5, 98.7) and 99.3% (95% CI 99.2, 99.4). The true negative rate or specificity was 91.8% for approach and 91.7% for carry. The true positive rate or sensitivity was 99.4% for approach and 99.7% for carry. For the evaluation of the digging classifier, the independent dataset contained the frames of seven annotated videos with in total 9476 frames (digging present = 44.1%; Table 2). The digging classifier accuracy was 85.0% (95% CI 84.6, 85.5), with a sensitivity of 81.8% and a specificity of 90.8%.

**Table 2:** Confusion matrices of manual and automated classification of retrieval status and maternal approach, carry and digging behaviors.

	Manual behavior absent (0)	Manual behavior present (1)
**Approach**		
Automated behavior absent (0)	24,676	233
Automated behavior present (1)	157	2618
**Carry**		
Automated behavior absent (0)	26,155	120
Automated behavior present (1)	76	1333
**Dig**		
Automated behavior absent (0)	14,412	925
Automated behavior present (1)	3218	9129

Retrieval status	Manual not retrieved (0)	Manual retrieved (1)
Automated not retrieved (0)	16	2
Automated retrieved (1)	6	36

Besides the frame-by-frame validation of these behaviors, the total count and duration of each behavior per video were validated using Pearson correlations. Table 3 shows the correlation matrices of maternal approach, carry and digging behaviors scored with three different methods: manual with no defined ROI, manual with defined ROI and our proposed automated scoring with ROI. Correlations were moderate to high (r = 0.51–0.90) between all scoring methods. Moreover, scoring with defined ROI showed high internal correlations for approach, carry and dig respectively (r = 0.62;0.77;0.90). These results suggest that defining an ROI has substantial impact on the PRT output. Predefined ROI are currently not used in PRT analysis, leading to a possible bias. Furthermore, the high correlations between both manual and automated scorings with ROI indicate that our automated model is appropriate for the estimation of the included maternal behaviors.

**Table 3:** Correlational matrices of maternal approach, carry and digging behaviors scored with three different methods: manual with no defined ROI, manual with defined ROI and automated scoring with ROI.

	Manual_noROI	Manual_ROI	Automated_ROI
**Approach**			
Manual_noROI	1.00	0.88	0.85
Manual_ROI	0.88	1.00	0.90
Automated_ROI	0.85	0.90	1.00
**Carry**			
Manual_noROI	1.00	0.64	0.51
Manual_ROI	0.64	1.00	0.62
Automated_ROI	0.51	0.62	1.00
**Dig**			
Manual_noROI	1.00	0.55	0.62
Manual_ROI	0.55	1.00	0.77
Automated_ROI	0.62	0.77	1.00

### Manual pup retrieval estimations

Raw retrieval videos were observed and categorized manually into “pup retrieved” or “pup not retrieved” classes. A pup trial was classified as retrieved as the mother carried it back to the nest^38^. In these videos the nest ROI was not visible. Successful retrieval trials were labeled ‘1’ and unsuccessful retrieval trials were labeled ‘0’. Additionally, the time was manually estimated for when the pup was carried back into the nest. Unsuccessful retrieval trials are assigned the maximum trial time (here: 90 s).

### Automated pup retrieval estimations

**Figure 2:**
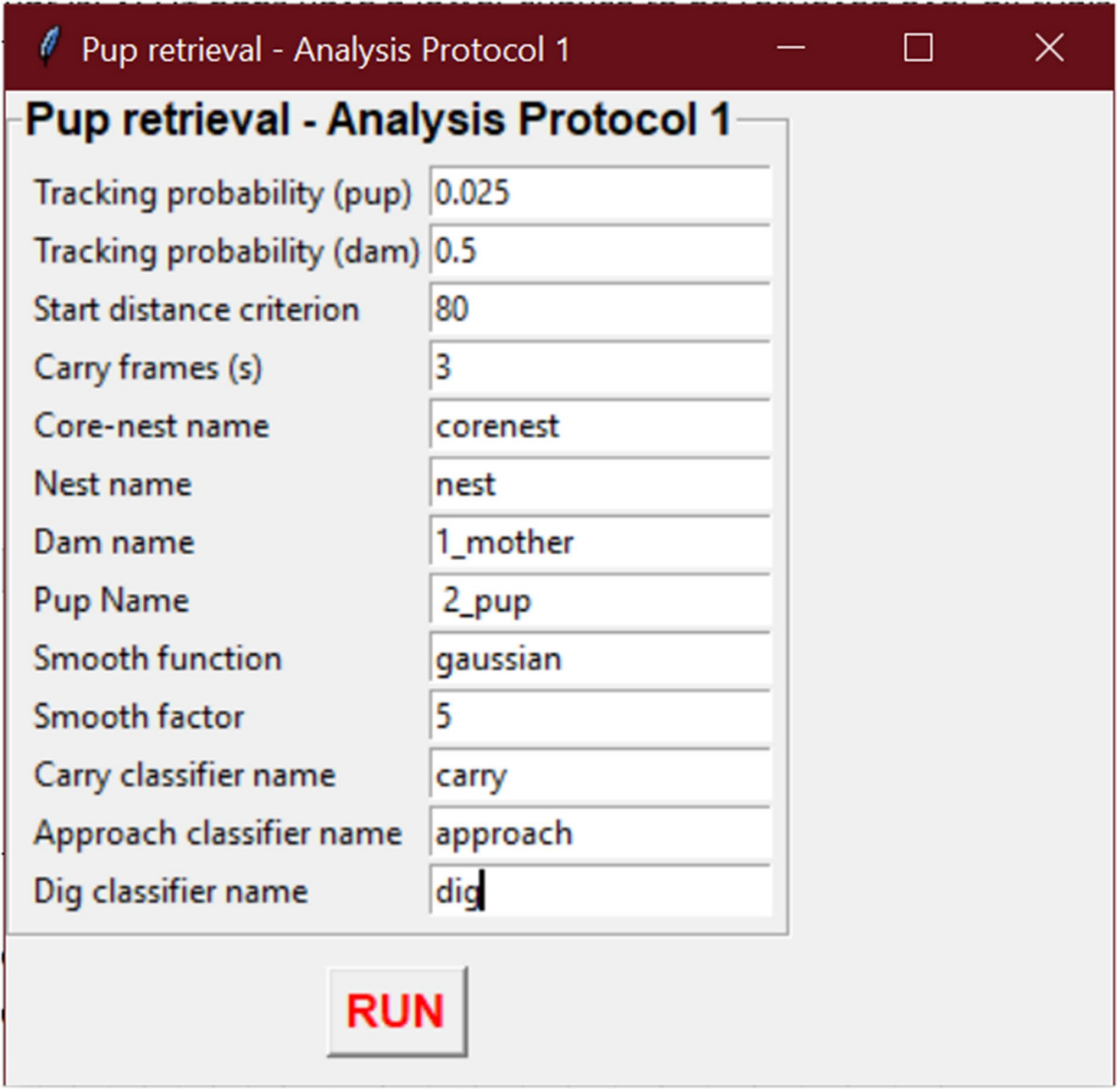
Pup retrieval analysis add-on in SimBA. A new module was built into SimBA for the automated analysis of the pup retrieval test, while performing a post-analysis quality control. Here, some thresholds are specified to resolve potential problems in tracking accuracies affecting the PRT outcome. The output provides the estimated retrieval success (retrieved vs not retrieved), time of retrieval and latency, total duration and counts for each behavior (carry, approach dig).

In SimBA, an add-on was created to evaluate results of the automated pup retrieval test, taking into account eventual tracking inaccuracies (Fig. 2). Here, retrieval is labeled as successful if at least one body part of a pup is present in the nest ROI and carry behavior was observed in the three seconds before entering. Similar as in the manual estimations, successful or unsuccessful retrieval events are scored respectively ‘1’ and ‘0’. Moreover, the output gives the estimated time of retrieval as well as the latency, total duration and counts for each behavior (carry, approach and dig).

### Performance evaluation of automated retrieval estimations

The prediction results created with SimBA were compared to the manually scored videos. Using the Caret package in R^38^, confusion matrices were obtained and analyses (Table 2). The accuracy of the retrieval success was estimated at 86.7% (95% CI 75.4, 94.1). The sensitivity and specificity of the predictions were respectively 72.7% and 94.7%. Next, manual time estimations and the predicted time estimations were correlated with r = 0.72. The prediction errors were examined in detail and could be classified into two types: (1) “boundary errors”, when the automated prediction was generally good, but the discrepancy with the manual estimation was related to ROI definition; (2) “Nest shift”, when the pup was predicted to be retrieved due to a moved nest but was without any doubt not retrieved in the nest in reality. Of the eight videos with a prediction error, two errors were explained by a shifted nest and six by boundary errors.

### PRT protocol, tracking models and behavioral classifiers

Our standardized PRT protocol can be found in Supplementary File 5, whereas our annotated images and videos, tracking models and behavioral classifiers are available on: 10.17605/OSF.IO/RWHTD. This protocol and these models are made available to the scientific community to ensure a more standardized analysis and more translatable results across researchers. Although our models and behavioral classifiers were constructed for specific settings (e.g. C57BL6J mice, home cage), they can be enlarged with their own data to obtain working models for different settings, as explained in our protocol (Supplementary File 5).

## Discussion

Pup retrieval is an essential feature of maternal care in mice, and PRT performance has consequently been used to study parental behavior in various research applications. The present paper proposes a novel, automated machine learning-based procedure to assess PRT performance, and identify performance variables in recorded PRT video files. Although our procedure uses machine learning, only basic equipment is required in combination with easy-to-use, open-access software. We used DLC to construct an accurate model for tracking a dam and one isolated pup synchronously. The model was able to track both the mother and the pup with high accuracy (mean body part tracking error between 2.2 and 4.5 mm). DLC has been used by others to track user-defined features on interacting animals, precisely and simultaneously, in simple and complex setups^37,39^. However, to date, most available software cannot be used to track a dam and her pup simultaneously, and track multiple body parts on each animal. The lack of such a tool has hindered the analysis of dyadic dam-pup interactions and manual scoring has remained the only possibility to quantify behaviors^28^. Manually annotating every frame in a one-hour video has been shown to require 22 working hours^37^. Additionally, manual scoring has been confounded by observer bias and observational drift, and had low reproducibility.

Manually scoring of successful pup retrieval is complicated by imprecise operationalization of retrieval success. We defined “successful retrieval” as “the experimental female […] retrieves the displaced pup back to the nest”, based on Marlin and colleagues^12^. However, the nest is not a predefined area, and it therefore depends on opinion whether “maternal transport to the periphery of the nest” is enough to label the trial successful. As mentioned, this ambiguity of underlying rules increases the chance of observer bias and complicate reproducibility and reliability^29,30,32,33^. The use of pre-defined ROIs during manual scoring already increased confidence in retrieval estimations, and we showed that our automated procedure yielded comparable results to manual scoring. Retrieval success was estimated with an accuracy of 87% (Table 2). One limitation, however, is that the nesting site is predefined and therefore fixed, but a mother may actually move the nesting site during the trial, and thus inflate retrieval scores. It might therefore be interesting to automate ROI definition in the future also.

Using automated behavioral classification, we were able to predict “maternal approach”, “carry” and “digging” with accuracies of 98.6%, 99.3% and 85.0%, respectively. These results are in line with studies using similar tools^31,40^. Further, the time estimated by our three classifiers for every behavior over the trials was highly correlated between automated and manual scoring for “approach” (r = 0.85–0.88), “carry” (r = 0.51–0.64) and “dig” (r = 0.55–0.77). These results indicate automated scoring yields comparable results to the golden standard of manual scoring. Important characteristics for transferability of behavioral classifiers are whether the features are position or rotation dependent^37^. SHAP-analysis (data not shown) showed that our “carry” and “approach” classifiers are grossly explained by a combination of dam-pup proximity and simultaneous movement, making them more likely to be transferable. However, “digging” was mostly explained by maternal position relative to the nest and core nest, indicating that “digging” might be less transferable. Unfortunately, we cannot assess how well these classifiers would perform in a different data space. Transferability of a constructed model to a new data space that looks different from previously encountered data has been a serious obstacle in deep learning approaches^41^. Here, we maximized transferability of the dam-pup model by avoiding to over-fit the network, or over-standardize cage environment (e.g., nest composition or camera position^42^). However, only recordings of black C57BL6J dams retrieving their pups on P5 in the home cage were used in the present study, but researchers often use different subjects (e.g., white CD-1 mice), different developmental ages^43^ or different test environments (e.g., T-shaped extension to the home cage^8,44^). A step-by-step protocol is provided in Supplementary File 5 for transparency and reproducibility, explaining how to expand the basic neural networks. Operational definitions of behavioral classifications are provided in Supplementary File 4, Table S4. By sharing all established datasets and models on OSF (10.17605/OSF.IO/RWHTD), the scientific community can achieve more generic models.

Typically, PRT read-outs have been limited to parameters such as “retrieval success” and “time to retrieve”. Sequential underlying behavioral components may be ignored by approaching maternal retrieval as a single behavior. However, retrieval comprises “maternal perception of pup distress”, “search” as well as “approach” components, and transporting the pup back to the nest^28^. By taking separate behaviors into account, retrieval vs non-retrieval can be more accurately analyzed (for example, by an inability to locate the pup). While the number of animals and workload of sampling stays equal, researchers can answer a much wider range of questions by taking these behaviors into account. In its current form, our automated PRT procedure included only on maternal “approach”, “carry” and “digging”, but other behaviors such as “nursing”, “pup licking”, “self-grooming”, and “disruptive digging on the pup” might ethologically relevant as well^45^. The flexibility of the used software allows to include other behavioral parameters, and the advantage of working with open-access software is that new features can be readily implemented^46^. As a final note, the use of unsupervised neural networks might be an interesting future direction. In contrast to supervised ones, unsupervised neural networks are not trained on data annotated by human researchers, which could even further avoid human bias, and identify behavioral components that relate to specific brain activity^37^.

## Material and methods

### Animals and ethics statement

Breeding pairs of primiparous C57BL/6JRj mice (8–10 weeks old) were purchased from Janvier Labs (Le Genest-Saint-Isle, France). Adult mice were group-housed and maintained for time-controlled breeding in standard type II cages. Males were only present in the home cage the night of mating and females were housed individually from gestation through weaning (P28). Mice were kept at 12/12 h light–dark cycle (lights on at 7 A.M.), water and food ad libitum, conditioned rooms (22 °C, humidity 30%). On day of birth (P0), pups were sexed and nests were reduced to 6 pups with a balanced male:female ratio. All animal procedures were approved the Animal Ethics Committee of KU Leuven (P028/2018), in accordance with European Community Council Directive 86/609/EEC, the ARRIVE guidelines and the ILAR Guide to the Care and Use of Experimental Animals.

### Pup retrieval test setup and protocol

Dams were tested on P5 and were transported to the test room at 8 A.M. PRT trials were performed between 9 and 10 A.M. A detailed step-by-step protocol can be found in Supplementary File 5. The PRT test was performed in the home cage inside a Styrofoam box to create a visually isolated environment. A Foscam C2 IP-camera (EUport, Wageningen) was connected to a laptop (Windows 10 as operating system) using an online interface and set at a height of 50 cm above the setup to record from an overhead camera perspective. Recording settings were as follows: a resolution of 1280 × 720 pixel; 10–30 frames per second (fps) and room lighting. Pups were placed in a clean glass cup, which was preheated to 35 °C using a heat pad. Every dam performed six semi-randomized retrieval trials (i.e. 3 male and 3 female trials) with a maximum time of 90 s per trial. A trial started as the mother was in the nest and a pup was placed in the most distant corner relative to the nest. If the pup is not retrieved within 90 s, the pup was placed back into the core of the nest.

### Image pre-processing

Video recordings were pre-processed using SimBA. First, spatial dimensions were cropped to fit the upper corners of the home cage (Fig. 1d). Second, videos were shortened to create separate trial videos (*e.g.* Mother-ID_Trial1). The start of the video was the first frame after placing the pup in the corner and where the researcher’s hand is not visible anymore. The end of the video was determined as 90 s after the first frame. Third, greyscale was applied to the videos. Finally, videos were formatted to .mp4 video format.

### Automated body part tracking using DeepLabCut

Pose-estimation (tracking) data, which is necessary to create features in SimBA, forms the basis of the accurate classification of behavioral patterns in video recordings^31^. We defined a minimalist 14-body part pose configuration for the mother and pup together, necessary to classify different maternal behaviors. DeepLabCut 2.1.10.4^35^ was used to develop a PRT mother–pup tracking framework. From 38 single trial video recordings, frames were extracted for labeling using k-means clustering. In total 592 frames were labeled as in Fig. 1a and Supplementary File 1 Table S1. Of these labeled frames, 95 percent were used for model training, whereas the remaining 5 percent was used to evaluate network performance. The mother–pup network was trained using ResNet-50 with a batch size of 4 images via Google Colaboratory (https://colab.research.google.com/).

### Automated behavioral classification in SimBA

For maternal approaching, carrying and digging, random forest classifiers were constructed in SimBA. In total, 27,683 frames of seventeen video recordings of PRT behavior were extracted and manually annotated for each of the three behaviors. To classify carry and approach, a subset of 10,157 frames from ten videos were used to construct classifiers. The digging classifier was constructed using 18,207 frames from ten different videos. Operational definitions can be found in Supplementary File 2 Table S4. All models were built with the following settings: n_estimators = 2000, RF_criterion = entropy, RF_max_features = sqrt, RF_min_sample leaf = 1, and random undersampling (approach = 8.5, carry = 16, digging = 2). A 75:25 ratio was used to split the annotated frames into a train:test dataset. Discrimination thresholds and minimum durations can be found in Table 1.

### Manual PRT scoring and behavior detection

Raw retrieval videos were classified ‘1’ if retrieval was successful and ‘0’ if retrieval was unsuccessful. A pup trial was classified as retrieved as the mother carried it back to the nest. In these raw videos the nest ROI was not visible. Next, the latency to being retrieved was manually estimated per video and unsuccessful retrieval trials are assigned the maximum trial time (here: 90 s).

### Automated pup retrieval estimations

In SimBA, an add-on was constructed to assess pup retrieval test performance. Here, retrieval is scored as successful if at least one body part of a pup is present in the nest ROI and carry behavior was observed in the three seconds before entering. Similar as in the manual estimations, successful or unsuccessful retrieval events are scored ‘1’ and ‘0’ respectively. Moreover, the output gives the estimated time of retrieval as well as the latency, total duration and counts for each behavior (carry, approach and dig).

### Computer software and hardware

A laptop equipped with an Intel Core i5-8350U CPU and 8 GM RAM was used for image annotations in DeepLabCut (http://www.mackenziemathislab.org/deeplabcut), behavioral classification in SimBA (https://github.com/sgoldenlab/simba) and data processing in R and Python. The mother–pup network was trained using Google Colaboratory (https://colab.research.google.com/).

## Supplementary Information

Supplementary Information.

## Data Availability

All our annotated images and videos, tracking models and behavioral classifiers are available on: 10.17605/OSF.IO/RWHTD. For further inquiries, please contact the corresponding author.
