# Author Correction: Automated procedure to assess pup retrieval in laboratory mice

**DOI:** 10.1038/s41598-022-06987-x

**Published:** 2022-02-14

**Authors:** Carmen Winters, Wim Gorssen, Victoria A. Ossorio‑Salazar, Simon Nilsson, Sam Golden, Rudi D’Hooge

**Affiliations:** 1 Laboratory of Biological Psychology, University of Leuven (KU Leuven), Leuven, Belgium; 2 Leuven Experimental Attachment Research Lab, KU Leuven, Leuven, Belgium; 3 Department of Biosystems, Center for Animal Breeding and Genetics, KU Leuven, Leuven, Belgium; 4 Department of Biological Structure, University of Washington, Seattle, WA USA

Correction to: *Scientific Reports* 10.1038/s41598-022-05641-w, published online 31 January 2022

The original version of this Article contained an error in the Discussion, where

“However, to date, neither DLC or other available software could be used to track a dam and her pup simultaneously, and track multiple body parts on each animal.”

now reads:

“However, to date, most available software cannot be used to track a dam and her pup simultaneously, and track multiple body parts on each animal.”

The original Article has been corrected.

