# Supplementary material for: Automated procedure to assess pup retrieval in laboratory mice
Source: Sci Rep. 2022 Jan 31;12:1663. doi: 10.1038/s41598-022-05641-w (PMC8803842; doi:10.1038/s41598-022-05641-w)
Supplement: Supplementary file 1 — Supplementary Information. [file 41598_2022_5641_MOESM1_ESM.docx]

Supplementary Files

**Supplementary File 1 Table S1. Operational definitions of body parts for labeling.**

**A mother-pup tracking framework was developed by labeling the following seven body parts on both the dam and the pup.**

| **Body part** | **Definition** |
| --- | --- |
| **Nose** | **Trivial; see Figure 1a** |
| **Ear left** | **Trivial; see Figure 1a** |
| **Ear right** | **Trivial; see Figure 1a** |
| **Center** | **Spinal location underneath the rib cage. This spinal location functions as a hinge as the mouse changes its direction of movement.** |
| **Spine 1** | **Spinal location where the shoulders are articulated to the spine.** |
| **Spine 2** | **Spinal location where the hips are articulated to the spine.** |
| **Tail base** | **Place on the body of the mouse where the tail starts.** |

**Supplementary File 2 Table S2. Pose estimation prediction errors (in pixels) with different p-cutoff values compared to the ground truth (manual annotations). For every DLC prediction a likelihood is calculated and a p-cutoff can be defined to filter unreliable predictions. The table shows that a higher confidence DLC prediction results in more precise feature position estimation.**

|  | **P-cutoff** | **None** | **0.1** | **0.5** | **0.9** | **None** | **0.1** | **0.5** | **0.9** |
| --- | --- | --- | --- | --- | --- | --- | --- | --- | --- |
| Individual | Bodypart | **Training dataset** | | |  | **Test dataset** | | | |
| Dam | nose | 4.91 | 4.91 | 4.31 | 3.02 | 15.59 | 15.59 | 12.61 | 7.22 |
| Dam | left ear | 4.19 | 4.19 | 3.37 | 3.05 | 12.95 | 12.95 | 7.71 | 4.6 |
| Dam | right ear | 3.88 | 3.85 | 3.78 | 2.92 | 12.69 | 12.69 | 7.48 | 3.47 |
| Dam | center | 3.89 | 3.89 | 3.43 | 2.95 | 10.94 | 10.94 | 9.34 | 8.57 |
| Dam | spine 1 | 4.22 | 4.22 | 3.76 | 3.1 | 9.96 | 9.96 | 9.84 | 5.56 |
| Dam | spine 2 | 4.09 | 4.09 | 3.63 | 2.91 | 11.02 | 9.06 | 9.14 | 7.57 |
| Dam | tail base | 4.44 | 4.41 | 3.95 | 2.83 | 13.6 | 10.44 | 9.38 | 6.48 |
| Pup | nose | 3.74 | 3.74 | 3.64 | 3.38 | 9.9 | 7.03 | 5.58 | 3.45 |
| Pup | left ear | 3.38 | 3.38 | 3.38 | 3.24 | 10.14 | 6.96 | 6.65 | 3.84 |
| Pup | right ear | 3.47 | 3.47 | 3.46 | 3.29 | 8.6 | 5.72 | 5.63 | 4.37 |
| Pup | center | 3.22 | 3.22 | 3.21 | 3.13 | 9.59 | 5.89 | 3.76 | 3.69 |
| Pup | spine 1 | 3.96 | 3.96 | 3.96 | 3.56 | 10.7 | 5.47 | 5.47 | 4.27 |
| Pup | spine 2 | 3.19 | 3.19 | 3.13 | 3.05 | 10.26 | 5.44 | 4.42 | 4.31 |
| Pup | tail base | 3.7 | 3.7 | 3.65 | 3.26 | 14.26 | 7.52 | 6.13 | 4.81 |

**Supplementary File 3 Table S3. Pose estimation errors with different p-cutoff values. A likelihood is calculated for every DLC prediction. A P-cutoff can be used to filter confident predictions. We calculated Mean error (mm) based on the test data set. The table shows that a higher confidence DLC prediction results in more precise feature position estimation.**

|  | **P-cutoff** | None | None | 0.1 | 0.1 | 0.5 | 0.5 | 0.9 | 0.9 |
| --- | --- | --- | --- | --- | --- | --- | --- | --- | --- |
| Individual | Bodypart | Mean error (mm) | sd | Mean error (mm) | sd | Mean error (mm) | sd | Mean error (mm) | sd |
| Dam | center | 4.8 | 0.3 | 4.9 | 0.8 | 4.1 | 0.3 | 3.8 | 0.3 |
| Dam | left ear | 5.7 | 0.4 | 5.8 | 1.0 | 3.4 | 0.2 | 2.0 | 0.1 |
| Dam | nose | 6.9 | 0.5 | 7.0 | 1.2 | 5.6 | 0.4 | 3.2 | 0.2 |
| Dam | right ear | 5.6 | 0.4 | 5.7 | 1.0 | 3.3 | 0.2 | 1.5 | 0.1 |
| Dam | spine 1 | 4.4 | 0.3 | 4.5 | 0.8 | 4.3 | 0.3 | 2.5 | 0.2 |
| Dam | spine 2 | 4.9 | 0.3 | 4.1 | 0.7 | 4.0 | 0.3 | 3.3 | 0.2 |
| Dam | tail base | 6.0 | 0.4 | 4.7 | 0.8 | 4.1 | 0.3 | 2.9 | 0.2 |
| Pup | center | 4.2 | 0.3 | 2.7 | 0.4 | 1.7 | 0.1 | 1.6 | 0.1 |
| Pup | left ear | 4.5 | 0.3 | 3.1 | 0.5 | 2.9 | 0.2 | 1.7 | 0.1 |
| Pup | nose | 4.4 | 0.3 | 3.2 | 0.5 | 2.5 | 0.2 | 1.5 | 0.1 |
| Pup | right ear | 3.8 | 0.3 | 2.6 | 0.4 | 2.5 | 0.2 | 1.9 | 0.1 |
| Pup | spine 1 | 4.7 | 0.3 | 2.5 | 0.4 | 2.4 | 0.2 | 1.9 | 0.1 |
| Pup | spine 2 | 4.5 | 0.3 | 2.4 | 0.4 | 1.9 | 0.1 | 1.9 | 0.1 |
| Pup | tail base | 6.3 | 0.4 | 3.4 | 0.6 | 2.7 | 0.2 | 2.1 | 0.1 |

**Supplementary File 4 Table S4. Behavioral operational classifiers: pup retrieval**

| Classifier | **Description** |  | **Start frame** |  | **Duration of behavior** |  | **End frame** |
| --- | --- | --- | --- | --- | --- | --- | --- |
| Approach | The dam moves towards the pup and sniffs it. |  | First frame when the dam elongates the body towards the pup to carefully sniff it. |  | Uninterrupted sniffing of the pup |  | First frame when the dam moves the head away from the pup or tilts head to take the pup into her mouth |
| Carry | The dam takes the pup into its mouth and transports it. |  | First frame when the dam tilts its head to take the pup into her mouth |  | Uninterrupted transporting of the pup using the mouth |  | First frame that the pup is no longer in the mouth of the mother. |
| Dig | The dam uses snout, front- or hindlimbs to displace bedding material. |  | 3.88 |  | Uninterrupted manipulation of the bedding material. |  | First frame that the mother is stop interacting with the bedding material |

Supplementary File 5: Protocol for automated PRT

Procedure

Preparation ● Timing 5 d

1. On the day of birth (postnatal day 0; P0), reduce the litter size to a maximum of 6 pups if necessary. Each nest should include at least two pups of each gender [47-48]. Nests are left undisturbed until P5 as researcher interference can affect cage dynamics in early-life [49-50].

! CAUTION Experiments using rodents must conform to local and national regulations. All animal studies and experimental procedures presented here were approved by the animal ethics committee of the University of Leuven (Belgium).

! CAUTION Some experimental interventions could cause interference before P5. Matched control procedures are always required, but it should be considered that parent-infant bonding might be affected in a more complex fashion. Therefore, conclusions should take possible interactions into account.

1. On the day of testing, clean the glass cup with 70% ethanol outside the test room to reduce odor cues and ensure proper disinfection. Prepare a pen, a recording sheet, balance and timer.

! CAUTION Check whether the video device contains enough memory for a video file of approximately 30 min (~200Mb/nest).

1. One hour prior to testing, transport the home cage with the mother and the pups to the test room for habituation to reduce stress.
2. While the mice are in the test room, silently prepare the set up. Firstly, preheat a heat pad to 35°C and place a clean glass cup onto it. Attach the thermometer probe to the bottom of the glass cup with a clean piece of tape. Secondly, set up the video sampling devices.

CRITICAL STEP The behavioral test room should be quiet, and temperature-controlled to avoid environmental confounds.

1. Set video recording parameters: our videos are recorded with a Foscam, top-down, 50 cm above the cage, greyscale at 10-30 fps and 1280x720 resolution.

CRITICAL STEP Behavioral classification is performed in SimBA with a temporal lower limit for approach, carry and digging (resp. 200, 500, 200 ms). We recommend recording at 25-30 fps.

1. Leave test room quietly.
2. At least five minutes prior to testing, place the first home cage to be tested into the Styrofoam box to create a visually isolated environment. Ideally, the mother should acclimatize for 60 minutes to the test setup. However, multiple nests might have to be subjected to the PRT and test time should be as standardized as possible [51].

! CAUTION Circadian fluctuations in general physiology and hormones should be minded. Therefore, testing should be fixed in time [51], and the experimenter should predefine an upper limit of testable nests per day.

Data sampling ● Timing 20-40 min per nest

1. Start video recording device.

CRITICAL STEP Before touching the pup, make sure to wear new gloves. Take some home cage bedding and rub it on your gloves.

1. Remove one pup from the core nest and place it in the heated glass cup. Record the sex of the pup. The pup stays in the heated cup until the mother is back on the nest. When the mother is back to the nest, place the pup at the most distant corner of the cage. Start timer.

CRITICAL STEP Do not rush while removing a pup. Make no sudden unexpected movements when your hand is in the home cage. Try to standardize the movement to place the pup in the cage (*e.g.* introduce the pup from the left side of the cage).

1. Trials have a fixed duration of 90s in which the mother can retrieve the pup. If the pup is not back in the nest within 90 seconds, place it back into the core of the nest. Between pups of the same nest, the glass cup is not cleaned since pup odors reduces stress in the pup.

! CAUTION Pups are not marked since odor is important in mother-infant recognition. Avoid if possible, although the same pup could be tested multiple times.

1. Repeat Steps 9-10 six times per mother, with three male trials and three female trials in pseudorandom order.
2. End the recording and name the collected video ‘Mother-ID_Date’.
3. Remove all pups from the nest and weigh them individually.
4. Take the home cage back to the animal room. In case of multiple nests, make sure to clean the glass cup outside the test room.

Data processing ● Timing

1. Pre-process video recordings using SimBA or another program of choice.
   - Crop the spatial dimensions to fit the upper corners of the home cage (Fig. 1d)
   - Shorten the videos to create separate trial videos (*e.g.* Mother-ID_Trial1). The start of your video should be the first frame after the ‘beep’ of the timer and otherwise after the first frame after placing the pup in the corner and the researcher’s hand is not visible anymore. The end of your video is 90 seconds after the first frame.
   - Apply greyscale if videos are sampled in color.
   - The video format should be either .mp4 or .avi.

CRITICAL STEP Make sure the experimenter’s hand is not in the videos since this would disrupt the model for tracking and behavioral classification.

Data processing: animal tracking without a GPU ● Timing ~ 60 min

1. Download our DeepLabCut tracking model from the OSF-page (<https://osf.io/rwhtd/>) in the folder ‘Dropbox: automatedPRT/Tracking – DeepLabCut’.
2. Organize the data as follows. On Google Drive, use your ‘My drive’-folder as the root directory to store the ‘PupRetrievalTest-CW’-folder (/content/drive/My Drive/PupRetrievalTest-CW).
3. In the PupRetrievalTest-CW’-folder, go to the ‘videos’-subfolder. Upload the videos you want to analyze.
4. In the OSF depository, go to the folder ‘Notebook’ and run the notebook ‘DeepLabCut tracking: CW-tracking model’ on Google Colab by pressing ‘Open in Colab’ → ‘Runtime’ → ’Change runtime type’ → select ‘GPU’.

| Box 1 \| Expanding the DLC model: ● Timing 1-2d |
| --- |
| Procedure  CRITICAL Pose-estimation can be adapted to experiments involving paternal, nulliparous females, brown-coat mice or mice with head posts (for optogenetics, electrophysiology and/or drug infusion).   1. After the application of the developed neural network, pose-estimation could be unsatisfactory due to:  - Jumps: one or more body parts jump a range of pixels from the last frame - Fitting: one or more body parts violate a state-space model fit to the time series.  1. Install DLC preferentially in Anaconda environment. (<https://github.com/DeepLabCut/DeepLabCut/blob/master/docs/installation.md>) 2. Download the ‘PupRetrievalTest-CW’-folder from the Google drive.   ! CAUTION The ‘PupRetrievalTest-CW’-folder should contain the .csv tracking files created in Step 24 of this protocol.   1. In this folder, open the config.yaml file and change the project_path to the correct directory on your computer. 2. Load the project in DLC and navigate to the ‘Extract outlier frames’-tab. Select 6-10 some videos with poor tracking and specify the video format (.mp4, .avi). Set shuffle to ‘1’ and the training set index to ‘0’. Define which algorithm you want to use and click ‘ok’.   ! CAUTION By default, 15 frames violating the chosen algorithm will be extracted. We advise to extract at least 90-150 extra frames (thus 6-10 videos) to expand the network.   1. In your Anaconda environment, you will be asked whether you want to proceed. Type ‘yes’ and press enter. A notification will appear in the Anaconda environment saying that the frames are in a subdirectory under labeled-data. 2. Go to the ‘Refine’ tab in DeepLabCut and click ‘launch’ to access these folders. Open the folders one-by-one and correct labels if necessary. The pose configuration is shown in Fig. 1a.   ! CAUTION Per animal, 7 points are annotated, which were chosen to have visually defined landmarks based on murine skeletal anatomy. ‘Center’ was defined as the spinal coordinate right underneath the rib cage, that functions as a hinge as the mouse changes its direction of movement. ‘Spine1’ and ‘Spine2’ were defined as the place of the spine where the limbs attach.   1. After the label refinement step, re-upload the project to your Google drive and change the project_path in config.yaml to: /content/drive/My Drive/PupRetrievalTest-CW 2. In the OSF depository, go to the folder ‘Notebook’ and run the notebook ‘DeepLabCut tracking: CW-expand model’ similarly to Step 18. 3. Run stages 1-3 as explained in Steps 19-21 of this protocol. 4. Create a new dataset by running the code in Stage 4 in Colab (this can take a few minutes). 5. Go to /PupRetrievalTest-CW/dlc-models/iteration-0/PupRetrievalTest-trainset95shuffle1/train and open the pose_cfg.yaml. Change the batch size to 4. 6. Run the cell under Stage 5 and the network training will start. As we are using a batch size of 4, the learning curve generally plateaus around 60k, i.e. your network will lose flexibility. Therefore, we advise to stop training at 65k iterations. 7. The last 40 snapshots will be stored in /PupRetrievalTest-CW/dlc-models/iteration-0/PupRetrievalTest-trainset95shuffle1/train. Check in that folder whether snapshots starting from 35k iterations are present. If they are not present, then they will be in the bin and need to be restored.   ! CAUTION Every snapshot has 3 corresponding files (.meta, .index, .data-00000-of-00001). It is important that all three files are restored.   1. Evaluate the created models as described in Box 2. 2. After identifying the best ranked model, proceed with Step 24. |

1. Run the cell underneath ‘Stage 1 - DeepLabCut installation in Colab’ by clicking the ►-button. This will take a few minutes.


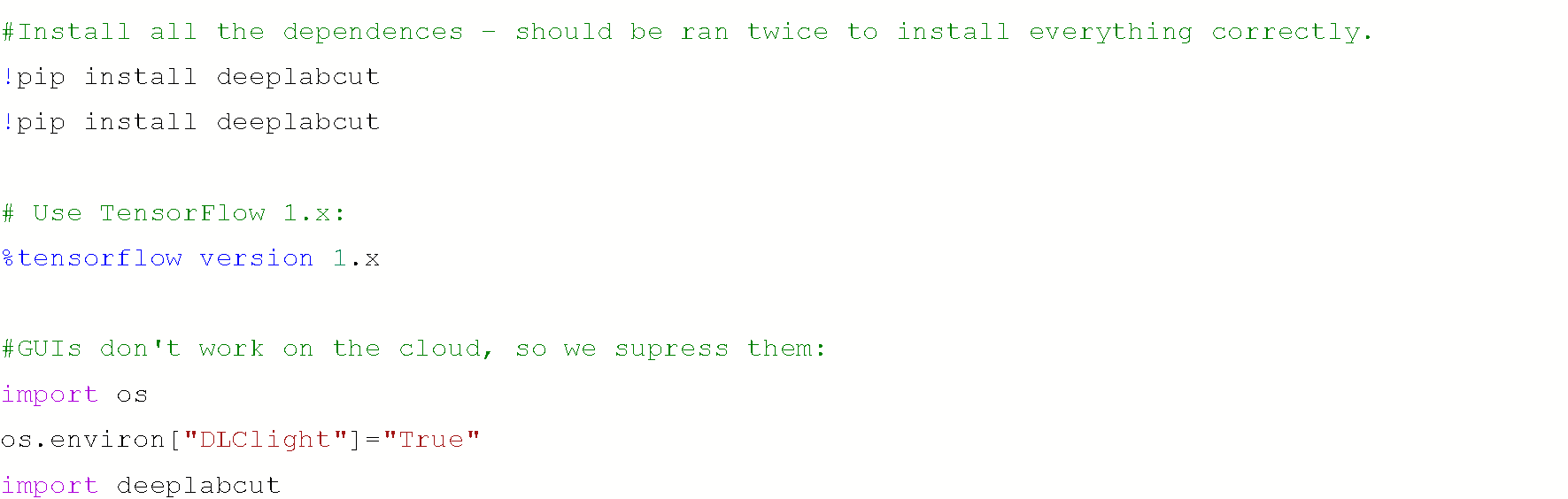


1. In stage 2, Colab will be linked to the Google Drive in which the ‘PupRetrievalTest-CW’ folder is stored. After running the cell, an authorization code is demanded. Click on the provided URL to obtain this code, which will lead to a browser window. Here, select the drive account where the PupRetrievalTest-CW’-folder was stored. After giving permission for access, an authorization code is received. Copy this code, paste it in the designated box and press enter.


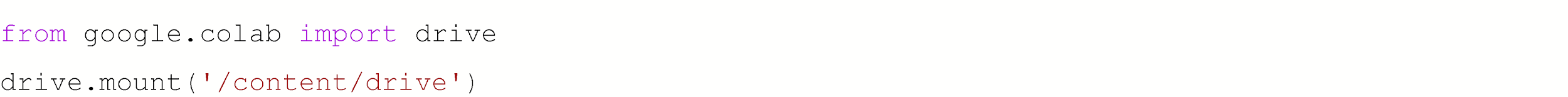


1. CRITICAL STEP Modify the parameters to be set in Stage 3. By default only the video format should be modified by changing ‘VideoType = ‘ to the format of the videos you want to analyze.


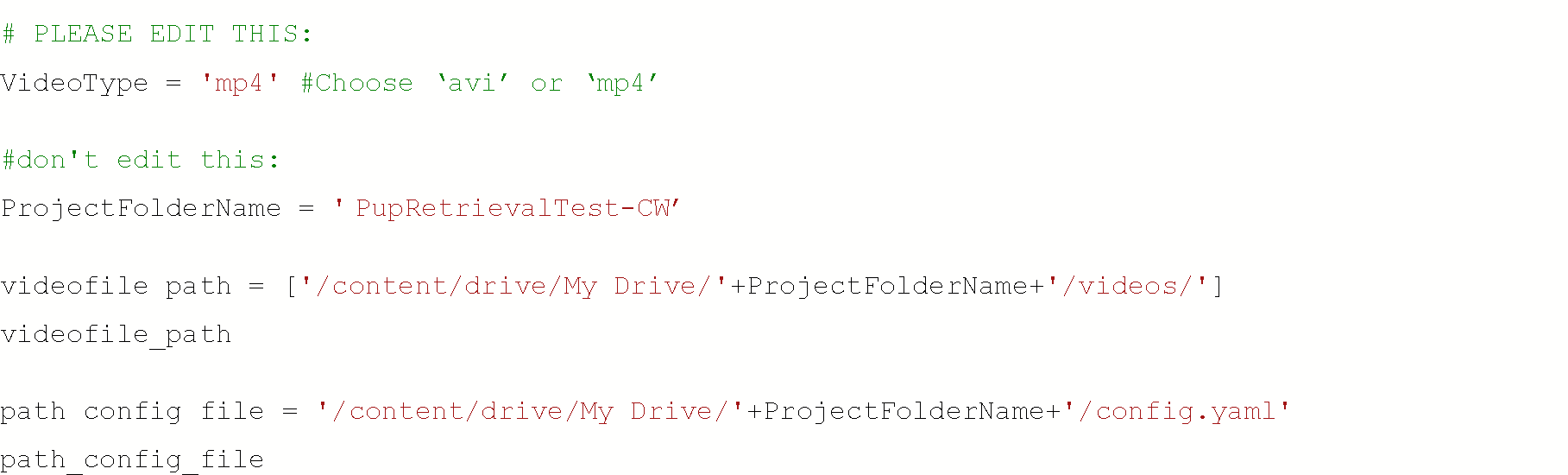


1. CRITICAL STEP The function in Stage 4 analyzes the novel video. The results are stored in a .hd5 and .csv files in the same directory as the video.


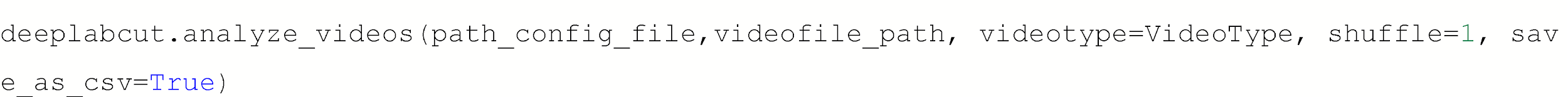


1. CRITICAL STEP The last function in Stage 5 is for visualization, which creates videos in .mp4 format with the predicted features. These are also stored in the same directory as the original video. The algorithm should be able to track the animals correctly when they are not covered by the nest.


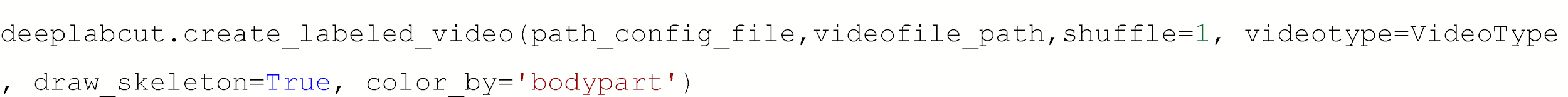


CRITICAL STEP Keep in mind that the following steps include outlier corrections. In the folder ‘PupRetrievalTest-CW/videos’, our labeled videos are included as an example of an example of good tracking with outliers.

CRITICAL STEP In case that the tracking is unsatisfactory, three solutions are possible:

- Features are being tracked on the subjects although they are not stable (jumps in tracking). We advise to use the jump algorithm in the label refinement option in DeepLabCut as explained in Box 1.
- Features are being tracked but not on the subjects (underfitting of the tracking model). We advise to use the fitting algorithm in the label refinement option in DeepLabCut as explained in Box 1.
- Features are not being tracked. We advise to create an entirely novel DeepLabCut tracking dataset. All parameters we used for our model are available on the OSF directory in the document ‘DeepLabCut_parameters’. Annotate approximately 500-700 frames and resume this protocol with Step 6 of Box 1.

1. Download all .csv files onto your computer.

| Box 2 \| Evaluating a new or expanded neural network in DLC ● Timing 60 min |
| --- |
| Procedure  CRITICAL Pose-estimation can also be adapted to experiments involving paternal, nulliparous females, brown-coat mice or mice with head posts.   1. Evaluate the network by considering the pixel error for all body parts. First, change ‘p-cutoff’ in the config.yaml to 0.1. Here, choose a model that is relatively low before p-cutoff, especially in the test data set. Further, the discrepancy between with or without cutoff pixel errors should be relatively stable since the model should be certain of its pose-estimations without high error rate. 2. Again, evaluate the networks for all body parts with a p-cutoff of 0.5. There needs to be a discrepancy between the with or without cutoff values, since 0.5 calculates pixel errors for relatively high probability pose estimations. 3. Evaluate the network by only taking the pixel error of the nose of the mother into account with a p-cutoff of 0.1. 4. Combine the results of these evaluations to choose the best model. Create labeled videos to see whether performance is satisfying. |

Data processing: behavioral classification using SimBA ● Timing

1. Download the SimBA folder on the repository (<https://osf.io/rwhtd/>) in the folder ‘Dropbox: automatedPRT/Behavioral_classification – SimBA’.
2. In SimBA, load the project_config.ini file in /PupRetrievalTest_CW_BehavioralClassification/project_folder. The ‘Load project’- window in SimBA will now open.
3. In the ‘Load project’- window continue as follows. Import the videos to analyze and import tracking data (.csv) files created with DLC.
4. Proceed to the tab ‘Video parameters’ and in the ‘Known distance (mm)’ box fill in ‘267’, if you used a type II animal cage. We use the length dimension of the top since the bottom part is covered with bedding. Then click ‘Autopopulate table’ and ‘Set video parameters’. On the right, you see the column ‘Get coord’ with every video in your folder. You can click on the video and the first frame of your video will pop up. Double-click (left button) on the cage tag hinges (Fig. 1c) and its parallel over the length dimension. Follow the instructions in SimBA.

! CAUTION SimBA provides the option to duplicate this distance over all included videos. Only do this if certain there are no differences in angle or cropping.

1. Proceed to the tab outlier correction. Go to settings and use for each animal the ‘Nose – Spine1’ body parts to perform outlier correction. We use 2.5 and 4 as movement and location criterions, respectively. Use the more robust median for eventual outliers.
2. In the tab ‘ROI’, define ROI shapes by adding 1 circle and 1 polygon and click ‘Show Shape Definitions Table’. Name the circle ‘corenest’ and the polygon ‘nest’. Do not enter a number in the radius box after circle. Click ‘Set Shape Definitions’ and a ‘ROI table’-window with all videos will pop up. We advise to draw ROIs for each file individually since these are important for outcome variables. For the ROI ‘corenest’, double click in the center of where your pups are laying together in the nest, and secondly click the outer border of where pups are located. Define the ROI ‘nest’ by double clicking on the borders of the nest with approximately a 15 mm margin (Fig 1d, example).
3. Click ‘Analyze ROI’ data and go to the tab ‘Extract features’. First, run the ‘extract features’ button and afterwards ‘Append ROI data to features’. A window will pop up asking which body parts to use. For the mother choose ‘Nose’, and for the pup choose ‘Spine1’.
4. Proceed to the tab ‘Run machine model’ and under the section Run Machine Model, then click on ‘Model settings’. A ‘Select model to run’-window will open. Click ‘Browse file’ and per classifier select the correct .sav-file in (…)/PupRetrievalTest_CW_BehavioralClassification/models/generated_models. The threshold for the behavioral classifiers approach, carry and dig are respectively 0.47, 0.47 and 0.24. Minimum bout lengths are 500 ms for approach and 1000 ms for dig, and 200 ms for carry. Click ‘Set model(s)’ and in the ‘Run machine model’ tab, click ‘Run RF Model’.
5. Go to the tab ‘Add-ons’ and click ‘Pup retrieval – Analysis Protocol 1. Use the default parameters as shown in Figure 2 and click run. In the folder (…)/PupRetrievalTest_CW_BehavioralClassification/project_folder/logs, a .csv-file named ‘Pup_retrieval_date’ is created. This file contains the test output including ‘frame to retrieval’, ‘latency to retrieval’, Boolean value whether pup was retrieved (0-1), Boolean value whether mother retrieved pup into core nest (0-1); and per behavioral classifier: the total time spent on the behavior before retrieval, latency to first event, number of events before retrieval, mean duration and mean interval duration.

Troubleshooting

Troubleshooting advice can be found in Table [1](#_qsh70q).

| Table 1 \| Troubleshooting table | | | |
| --- | --- | --- | --- |
| Step | Problem | Possible reason | Solution |
| 10 | Deviant pup/nest weight on P5 (mean-2SD) |  | Do not include in analysis |
| 9 | Interference during test procedure | Unplanned event (such as a loud noise) occurred while sampling data | Do not include trial in analysis |
| 21 | Pose-estimation model does not fit on the subjects in the video. In the labeled video, features are repeatedly plotted on the same spot in the margin of the cage | An object is present at the side of the cage | Crop the video to exclude the object, if possible. |
| 21 | Pose-estimation model does not fit on the subjects in the video | Video is recorded too far or too close inside the cage, or video is not sampled centered top-down | This cannot be solved for the current pose estimation model. You can expand the neural network using Box 1 |
| 21 | Pose-estimation model does not fit on the subjects in the video | The test animal is looking different from a C57bl6j mother used to create the pose estimation model or the pup is smaller/larger than P5 C57bl6j pups | Expand the neural network using Box 1 |
| 27 | Pose estimation appears unstable after outlier correction | The criterion value for location and/or movement outlier correction is too small | Re-run outlier correction with a higher criterion value |

Timing

Step 1, Stage I, sexing pups and culling: ~5 min/nest

Steps 2-5, Stage II, preparation of test including transport and habituation: ~70 min

Steps 6-10 , Stage III, pup retrieval test sampling: variable, ranging from 20 to 40 min per nest

Step 11, Stage IV, pre-processing of video files: variable, approximately 5 to 10 min per video

Steps 12-13, Stage V, preparation of videos for analysis in Colab: 5 min,

Steps 14-19, Stage VI, preparation of Colab: 5-10 min

Step 20, Stage VII, video analysis in Colab: variable, > 1 min per video

Step 21, Stage VIII, video analysis in Colab: variable, ~ 1 min per video

Steps 23-25, Stage IX, preparation SimBA analysis: ~ 5 min

Step 26, Stage X, distance definition in SimBA: variable, ~ 5 to 10 min

Step 27, Stage XI, Outlier correction in SimBA: variable, < 1 min per video

Step 28, Stage XII, ROI definition in SimBA: variable, ~ 2 min per video

Steps 29-30, Stage XIII, ROI definition in SimBA: variable, ~ 30-60 min

Step 28, Stage XIV, Extract parameters: variable, ~ 10 min

Materials

Biological materials

- Breeding pairs

Pairs of primiparous C57BL/6JRj mice were purchased from Janvier Labs (Le Genest-Saint-Isle, France), and maintained for time-controlled breeding in standard type II cages. Males were only present in the home cage the night of mating and females were housed individually from gestation through weaning (P28).

- Laboratory-bred mouse pups on P5

For the test, each nest should contain a fixed number of pups with a balanced male:female ratio. On day of birth (P0), sex differentiation is based on the anogenital dark spot in males. Nests should be reduced to 6 pups with a preferable 3:3 male:female ratio [47-48].

! CAUTION Experiments using rodents must conform to local and national regulations. All our experiments were reviewed by the animal ethics committee of the University of Leuven (Belgium), in accordance with European Community Council Directive 86/609/EEC.

Reagents

- 70% (vol/vol) Ethanol solution

Equipment

- Clean gloves.
- Standard Type II cage, bottom dimensions 225 x 167 mm, top dimensions 267 x 208 mm and height 140 mm.
- Styrofoam box: 370 x 300 mm and height 330 mm.
- Stable heat pad: we use an IPower reptile heat pad with digital thermostat for small animals.
- Glass cup to place the pup in on the heat pad: we use a borosilicate 3.3 glass crystallizing dish (50 mm diameter, 30 mm height; VWR, cat. no. 216-0063).
- Balance (to weigh pups after testing)
- Stopwatch (to time maximum trial time)
- Tape to attach the thermometer probe of your heat pad to the glass cup.

Software

- Operating system: Windows (10).

! CAUTION SimBA is not compatible with other systems.

- Google drive
- Anaconda: free and open-source distribution of the Python programming language (<https://www.anaconda.com/>). SimBA is written in Python 3.6 (<https://www.python.org/>) and is not compatible with Python 2.
- SimBA: free and open-source toolbox available at <https://github.com/sgoldenlab/simba>. The code is written for Python 3.6.
- Microsoft Office Excel

Hardware

- Computer; The SimBA toolbox can be used on modern desktop workstations as well as laptops.
- Video camera: we use Foscam C2 IP-camera and online interface (EUport, Wageningen), at a resolution of 1280x720px and 10-30 frames per second (fps). The camera was fixed in the center of the Styrofoam box, approximately 50 cm above the floor.

Biological materials setup

- Animal housing

Adult mice (8-10 weeks old) were group-housed under standard housing conditions. Mice were kept at 12/12 hour light-dark cycle (lights on at 7 AM), water and food *ad libitum*, conditioned rooms (22°C, humidity 30%). A 14-day acclimation period before mating avoided transportation distress to affect the dams. Nests were transported to the test room at 8 AM and testing took place between 9 AM and 10 AM.

Equipment setup

- Testing setup

For testing, the home cage without grid is placed inside the Styrofoam box without lid to create a visually isolated environment. A camera is fixated 50 cm above the center of the floor of the Styrofoam box to record top-down. The heat pad is positioned next to the Styrofoam box with the glass cup placed on it. The thermometer probe should be taped to the bottom of the glass cup. The camera is connected to a laptop distanced approximately 1.5 m from the Styrofoam box. The balance is positioned between the laptop and the Styrofoam box.
